# Spatio-temporal home range of the dominant rodent species in Mabira central forest reserve, Uganda

**DOI:** 10.1186/s12862-023-02148-4

**Published:** 2023-08-21

**Authors:** James Ssuuna, Rhodes H. Makundi, Simon J. Chidodo, Moses Isabirye, Nsajigwa E. Mbije, Loth S. Mulungu

**Affiliations:** 1 Africa Centre of Excellence for Innovative Rodent Pest Management and Biosensor Technology Development, Sokoine University of Agriculture, Morogoro, Tanzania; 2 Department of Natural Resource Economics, Busitema University, Tororo, Uganda; 3 Department of Wildlife Management, Sokoine University of Agriculture (SUA), Morogoro, Tanzania; 4 Institute of Pest Management, Morogoro, Tanzania

**Keywords:** Tropical high forest, Disturbed habitats, Rodents

## Abstract

**Background:**

Rodents form the largest order among mammals in terms of species diversity, and home range is the area where an individual normally moves during its normal daily activities. Information about rodent home ranges is paramount in the development of effective conservation and management strategies. This is because rodent home range varies within species and different habitats. In Uganda, tropical high altitude forests such as the Mabira Central Forest Reserve are experiencing continuous disturbance. However, information on rodent home range is lacking. Therefore, a two year Capture-Mark-Release (CMR) of rodents was conducted in the intact forest habitat: Wakisi, regenerating forest habitat: Namananga, and the depleted forest habitat: Namawanyi of Mabira Central Forest Reserve in order to determine the dominant rodent species, their home ranges, and factors affecting these home ranges. The home ranges were determined by calculating a minimum convex polygon with an added boundary strip of 5 m.

**Results:**

Overall, the most dominant rodent species were: *Lophuromys stanleyi*, *Hylomyscus stella*, *Praomys jacksoni Mastomys natalensis*, *Lophuromys ansorgei*, and *Lemniscomys striatus*. *H. stella* dominated the intact forest habitat, while *L. stanleyi* was the most dominant both in the regenerating and the depleted forest habitats. *L. stanleyi* had a larger home range in the depleted forest, and the regenerating forest habitats, respectively. In the regenerating forest habitat, *M. natalensis* had a larger home range size, followed by *L. stanleyi*, and *L. striatus*. While in the intact forest habitat, *H. stella* had the largest home range followed by *P. jacksoni*. *H. stella*, *L. striatus*, *L. stanleyi*, *M. natalensis*, and *P. jacksoni* were most dominant during the wet season while *L. ansorgei* was relatively more dominant during the dry season. *L.* *ansorgei*, and *P.* *jacksoni* had a larger home range in the dry season, and a lower home range in the wet season. *H. stella*, *L. stanleyi*, *M. natalansis* and *L.striatus* had larger home ranges in the wet season*,* and lower home ranges in the dry season.

The home ranges of the dominant rodent species varied across the three habitats in Mabira central forest reserve (${F}_{(2, 15)}= 6.41$, $p = 0.000$).

**Conclusion:**

The significant variation in home ranges of the dominant rodent species in Mabira Central Forest Reserve depending on the type of habitat presupposes that the rodent management strategies in disturbed forest reserves should focus on the type of habitat.

## Introduction

Rodents form the largest order among mammals in terms of species diversity. A home range is the area where an individual normally moves during its normal daily activities. It varies depending on various factors such as sex and breeding period. In small mammals, males typically have home ranges that can be twice as large as those of females [1]. In rodents, home range size of males is always larger than that of females [2]. Reproductively active males maintain larger home ranges than females because they have to eat more food to acquire more energy for mating success [3, 4]. Home range size can also vary within species due to differences in the quality of habitat, distribution, and abundance of food, or population density. Increase of population density during the breeding period affects the degree of intersexual overlap of home range, and factors affecting resource availability and distribution (such as nature of habitat, season, etc) directly affect home ranges [2].

Natural forests always form conducive habitats for many small mammals [5]. Natural forests are forests always composed of mainly indigenous trees, and are conducive for small mammals because they form quality habitats. Changing environmental factors may affect the different forest habitats. Thus, impacting on the population dynamics of the different small mammal species [6]. Many Ugandan forests are undergoing threatening levels of destruction due to increasing human activities [7, 8]. This has continuously impacted on the forest ecosystems [9].

Mabira forest is a natural forest reserve (protected tropical high forest) with a diversified rodent community structure. Due to increased settlements in the forest and encroachment by the neighboring community from the surrounding big cities and towns (Kampala, Jinja, Mukono, Kayunga), the forest has been transformed into three distinct habitats, namely; the depleted forest, regenerating forest (young and colonizing forest), and intact forest (mature mixed forest with very limited disturbance) [10, 11]. Specifically, Namananga forest reserve represents the section of Mabira that is under natural regeneration with an average tree height less than 15 m., with one part of the reserve in a swamp dominated by *Learsia hexandra* and the forested expanse dominated by *Brousonetia papyrifera*, and small fields of cultivation and areas of human settlement. Namawanyi forest reserve is dominated by *Brosonetia papyrifera* with very few indigenous trees and is fringed by fields of cultivation. It is the reserve with very high levels of destruction with many sections completely depleted and turning into bushed grasslands/ bushed fallows, and always experience seasonal bush burning. Wakisi forest section represents the intact part with relatively limited levels of disturbance.

Information on home range size of different rodent species could be paramount in development of the effective conservation and management strategies of rodents in different habitats. However, for a number of natural forests in Uganda undergoing severe destruction, such information is scanty and lacking. We conducted this study to provide details on the home range of rodents in a tropical high forest located in central Uganda. The information generated may improve our understanding of the ecology of rodents in natural habitats, and provide a basis for developing effective management strategies for some of the selected species. Thus, in this study, it was hypothesized that home range sizes of dominant rodent species in Mabira Central Forest Reserve (MCFR) do not differ depending on rodent habitat type, and season**.**

## Materials and methods

### Study area and sampling sites

**Fig. 1:**
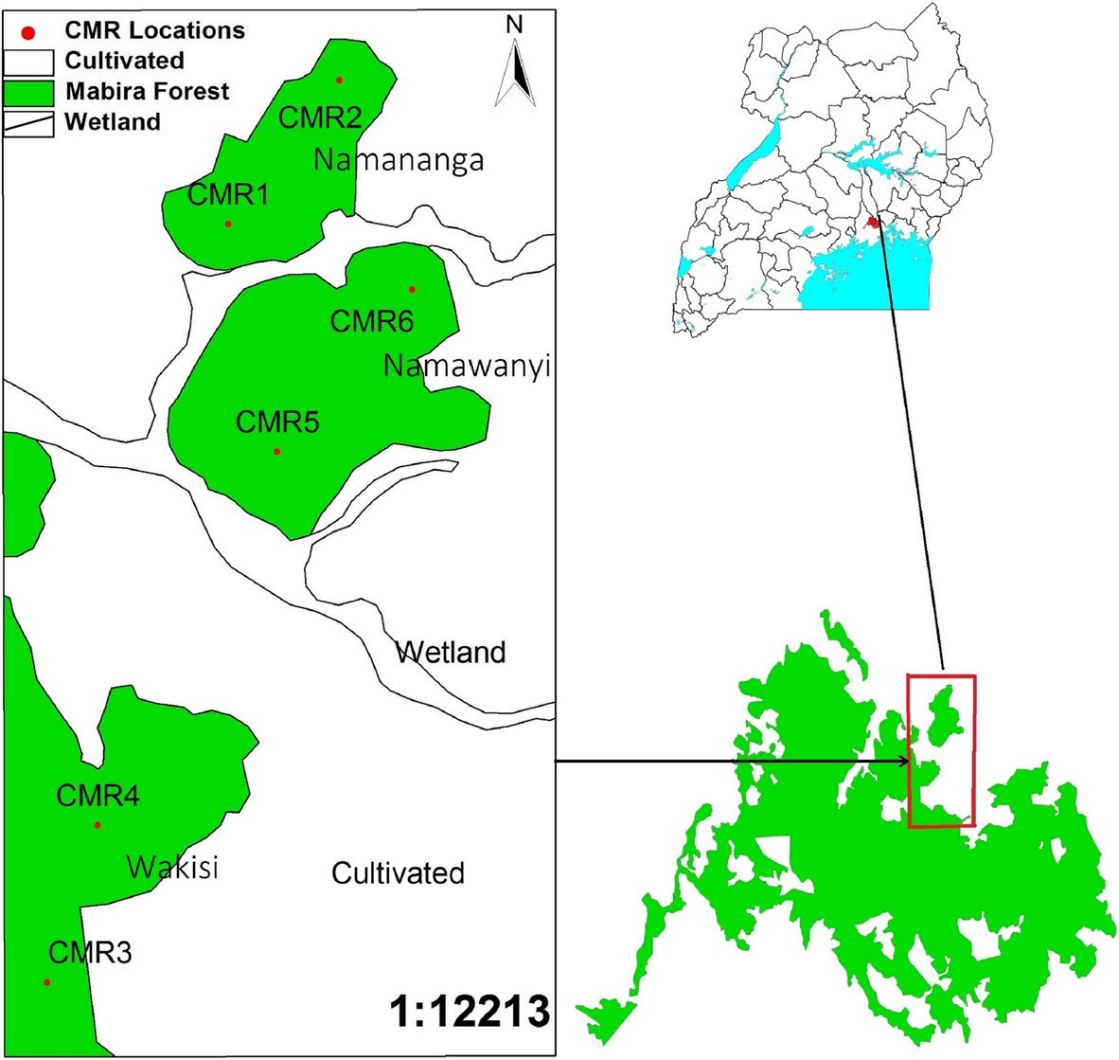
Map showing the study sites in Mabira forest (This is my own map generated using the GPS coordinates taken from each of the 3 study sites)

This study was carried out in Mabira central forest reserve. This reserve is found 54 km away from Kampala capital city at an altitude of 1070–1340 m a.s.l, average temperature of 26 °C, and covers three different districts: Mukono, Buikwe, and Kayunga districts. The area receives two main rain seasons: March to May (MAM) and September to December (SOND) [12, 13]. Due to continued forest disturbance, the forest has been reduced to three distinct habitats: the Intact part, regenerating, and the depleted part/bushed grassland. Specifically in this study, 3 habitats, were selected subjectively from the forest (Fig. 1): Wakisi forest section representing the intact part, Namananga forest representing the regenerating forest habitat, and Namawanyi forest, representing the depleted habitat [13]. The distance in between the intact and the regenerating forest habitat is approximately 8 km, while the distance between the regenerating and the depleted forest habitats is approximately 3 km, and the distance between the depleted and the intact forest habitat is approximately 5 km.

### Rodent trapping by capture mark release

Six grids were laid for live-trapping of rodents using CMR method for a period of 24 months (September, 2018 to August, 2020). Grids 1, and 2 were located in the regenerating forest habitat fields, spaced at a distance of 2 km away from the other grids, Grids 3, and 4 were set in the intact forest habitat fields with approximately 1.5 km between them, while grids 5 and 6 were laid in the depleted forest habitat fields with 2 km in between them (Fig. 1). 49 Sherman Live traps were set in each of the six grids of size 70 m by 70 m (with a 5 m boundary strip at each of the corners) each containing 7 parallel lines spaced 10 m apart, and 10 m between traps, each parallel line having 7 trapping stations. Trapping of rodents was done using Sherman live traps, each baited with a mixture of local ghee, peanut butter, ripe bananas, and maize grains. The traps were set for 3 consecutive nights on a monthly basis. Trap inspection was done early morning on each day of trapping [13, 14].

### Data processing and analysis

All captured rodents were carefully removed from the traps using a cloth bag, weighed using a Pesola balance, identified using morphometric measurements and recent literature [15], and thereafter given a unique identifier by toe clipping using a sterilized scissor and released at the same point of capture. In order to confirm the identified species, further analysis using deoxyribonucleic acid (DNA) was done at the Institute of Vertebrate Biology, the Czech Academy of Sciences.

Variables of interest for each animal trapped were: grid location and grid number, date, toe clipping code, species, sex, and body weight, maturity and breeding status. Composition of species over the study period was done using a statistical package StataIC12.0, and upon this, the dominant rodent species were identified as those with relatively higher species frequency.

Using PAST Statistics software, the Simpson Diversity Index (SDI) was estimated a measure of diversity in rodent species because it gives more weight to dominant species in a sample. The SDI was computed using the formula:

1 $$SDI=1-\frac{\sum n(n-1)}{N(N-1)}$$

where; *n* is the number of individuals of different species, *N* is total number of individuals of all the species.

The home ranges for the dominant rodent species were then determined by calculating a minimum convex polygon (MCP) with an added boundary strip of 5 m (half the distance between neighboring traps [1, 16, 17] using Ad habitat package in R software version 4.3. For all the analyses, all locations where an individual was captured were used for MCP estimation. To ascertain whether home ranges varied across habitats, and seasons, Analysis of variance (ANOVA) and the interaction plot were used. The level of significance was 5%.

## Results

### Species composition

A total of 1537 rodent captures were made in 24 months. Out of the number of rodents captured, 562, 632, and 343 rodent individuals were trapped in the intact, regenerating, and depleted forest habitats, respectively. These comprised of 13 rodent species: *Aethomys hindei (*Thomas, 1902), *Deomys ferrugineus* (Thomas, 1888), *Grammomys skuru (*Thomas & Wroughton, 1907), *Hybomys univittatus (*Peters, 1876), *Hylomyscus stella* (Thomas,1911), *Lophuromys stanleyi (*Verheyen, et al., 2007), *Lophuromys ansorgei (*de Winton, 1896), *Lemniscomys striatus* Linnaeus, 1758), *Mastomys natalensis* (Smith, 1834), *Mus bufo*, *Praomys jacksoni* (de Winton, 1897), *Rattus rattus* Linnaeus, 1758, and *Gerbilliscus kempii* (Wroughton, 1906). *H. stella* and *P. Jacksoni* dominated the intact forest (IF), *L. stanleyi* and *M. natalansis* dominated the regenerating forest (RF), while in the depleted (DF), *L. stanleyi*, *L. ansorgei*, and *L. striatus* were most dominant. Overall, *L.* *stanleyi* was the most dominant *natalansis* rodent specie with 315 individuals and the least dominant were *G.* *skuru*, *H.* *univittatus*, *R.* *rattus*, and *G.* *kempi* (Table 1).

**Table 1:** Rodent species captured during the study period in Mabira Central Forest Reserve, Central Uganda, year 2018–2020

	**Habitat**			
**Species**	IF (%)	RF (%)	DF (%)	Overall number (%)
*Aethomys hindei*	0	60 (9.5)	30 (8.7)	90 (5.8)
*Deomys ferrugineus*	20 (3.6)	2 (0.32)	0	22 (1.4)
*Grammomy skuru*	0	1(0.2)	0	1 (0.1)
*Hybomys univittatus*	1 (0.2)	0	0	1 (0.1)
*H ylomyscus stella*^b^	300 (53.4)	3 (0.5)	1 (0.3)	304(19.8)
*Lophuromys stanleyi*^b^	14 (2.5)	173 (27.4)	128 (37.3)	315 (20.5)
*Lophuromysansorgei*^b^	6(1.1)	79 (12.5)	83 (24.2)	168 (10.9)
*Lemniscomysstriatus*^b^	0	49(7.6)	77 (22.4)	126 (8.2)
*Mastomys natalansis*^b^	2 (0.4)	159 (25.2)	13 (3.8)	174 (11.3)
*Mus cf. bufo*	5(0.9)	62(9.8)	6(1.8)	73(4.7)
*Praomys jacksoni*^b^	214 (38.1)	32 (5.1)	3 (0.9)	249 (16.2)
*Rattusrattus*	0	3 (0.5)	2 (0.6)	5 (0.3)
*Gerbilliscus kempi*	0	9 (1.4)	0	9 (0.6)
Total Captured	562 (100)	632 (100)	343 (100)	1537 (100)

*IF* Intact forest, *RG* Regenerating forest, *DF* Depleted forest

^a^Numbers in brackets are percentage contribution of each species in a certain habitat

^b^Dominant rodent species

The regenerating forest habitat had the highest species richness, followed by the depleted and intact forest, respectively. The Simpson diversity index indicated that species diversity was highest in the regenerating forest habitats followed by depleted forest habitats and lowest in intact forest habitats (Table 2).

**Table 2:** Diversity Indices for rodents captured in the 3 habitats of Mabira central forest reserve, Uganda

Index	Habitat		
	Intact forest	Regenerating forest	Depleted forest
Species richness	8	12	9
SDI	0.57	0.82	0.75

*SDI* Simpson’s Diversity Index

### Home range of the dominant rodent species across habitats

Results in Table 3 show the home ranges of the dominant rodent species in Mabira central forest reserve. *L. ansorgei* had the largest home range size in the depleted forest habitat and a smaller home range in the intact forest. *H. stella* had a larger home range in the intact forest habitat as compared to the regenerating forest. Besides, *L. stanleyi* had a relatively larger home range in the regenerating forest habitat as compared to the depleted forest habitat. *M. natalansis* had a higher home range in the regenerating forest as compared to the depleted forest habitat. *L. striatus* had a larger home range in the depleted forest habitat as compared to the regenerating forest habitat. *P. jacksoni* had a larger home range in the intact forest habitat as compared to regenerating forest habitat (Table 3).

**Table 3:** Home ranges ($\mathrm{Home range}\pm \mathrm{standard error}$) of dominant rodent species in the different habitats

Species	DF	RF	IF
*Hylomyscus stella*	-	92.50±8.20	185.35±16.85
*Lophuromys ansorgei*	222.05±19.08	74.01±6.00	50.02±3.25
*Lophuromys stanleyi*	182.04±11.81	192.07±12.85	81.50±5.90
*Mastomys natalansis*	41.24±4.00	94.03±11.74	-
*Lemniscomys striatus*	200.00±9.54	66.67±3.18	-
*Praomys jacksoni*	-	54.78±9.54	164.37±28.61

**Fig. 2:**
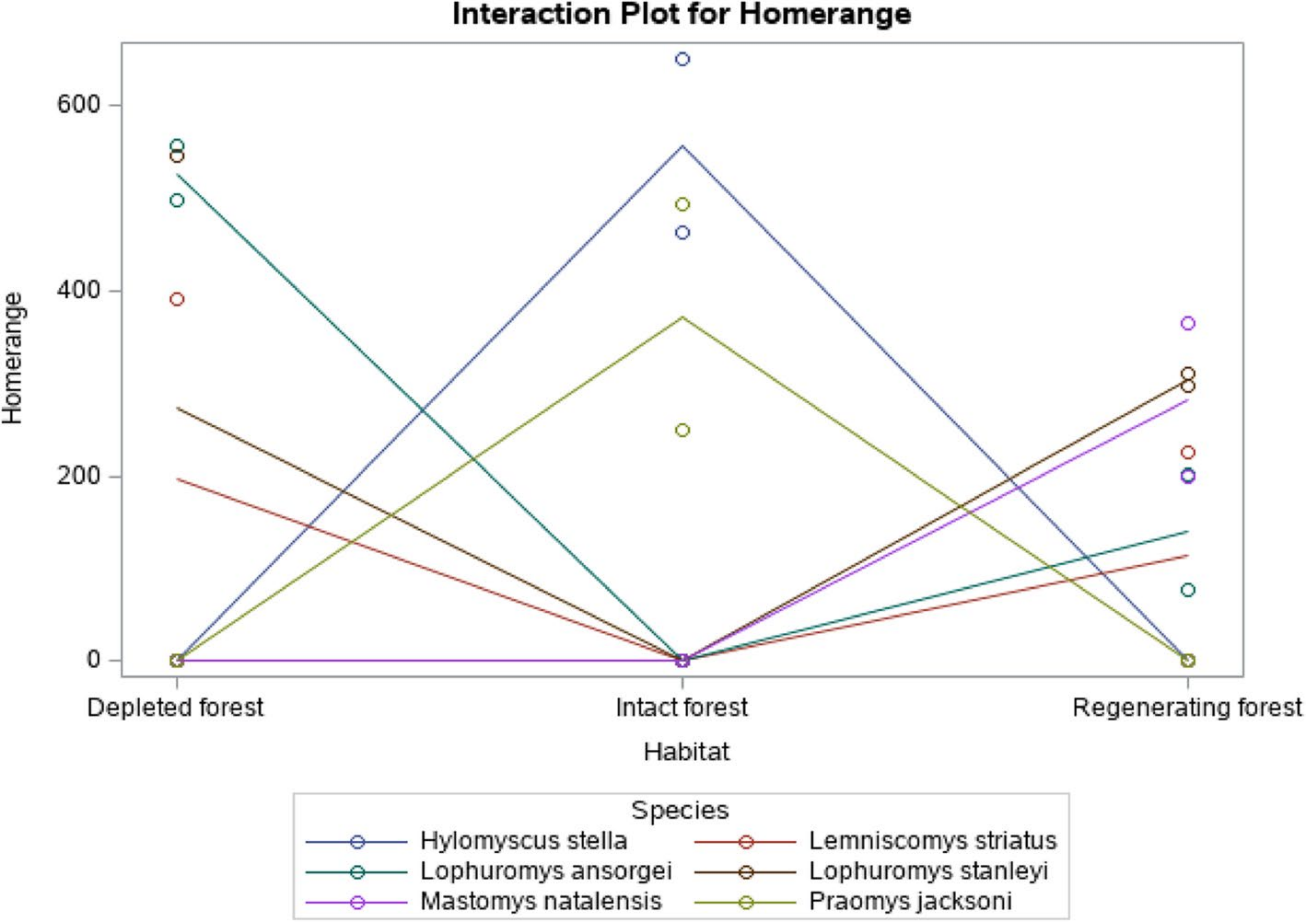
Home ranges of the dominant rodent species across the three habitats

Results in Fig. 2 indicate that *L. ansorgei* had the highest home range size in the depleted forest. This was closely followed by *L. stanleyi*, and *L. striatus*, respectively in the same habitat. In the regenerating forest habitat, *L. stanleyi* had a relatively larger home range size, followed by *M. natalensis*, *H. stella* and *L. striatus*. While in the intact forest habitat, *H. stella* had the largest home range followed by *P. jacksoni*, and *L. striatus*, respectively.

Further analysis indicated that home ranges of the dominant rodent species significantly varied across the three habitats (${F}_{2, 15}= 6.41$, $p = 0.000$) (Table 4).

**Table 4:** Analysis of variance based on home range by habitat

Source	SS	Df	MS	F	Prob > F
Between groups	254.20	2	127.10	6.41	0.000
Within groups	297.43	15	19.83		
Total	551.63	17			

### Distribution of dominant species by season

*H. stella*, *L. striatus*, *L. stanleyi*, *M. natalansis*, and *P. jacksoni* were most dominant during the wet season while *L. ansorgei* was relatively more dominant during the dry season (Table 5).

**Table 5:** Distribution of dominant rodent species by season

Dominant species	Season	
	**Dry**	**Wet**
*Hylomyscus stella*	104	200
*Lemniscomys striatus*	50	76
*Lophuromys ansorgei*	91	78
*Lophuromys stanleyi*	118	197
*Mastomys natalansis*	66	108
*Praomys jacksoni*	99	150

#### Dominant species home ranges across seasons

*L. ansorgei* had a larger home range in the dry season, and a lower home range in the wet season. This was followed by *P. jacksoni* with a larger home range in the dry season and lower home range in the wet season. In contrast, *H. stella* had a larger home range in the wet season and a lower home range in the dry season $.$ *L. stanleyi* had a larger home range size in the wet season and lower home range in the wet season. *M. natalensis* had larger home range size in wet season and lower home ranges in the dry season (Table 6).

**Table 6:** Home ranges (Home range±standard error) of different rodent species across seasons

Species	Season	
	**Dry**	**Wet**
*Hylomyscus stella*	153.9±26.14(104)	216.8±26.55(200)
Lophuromys ansorgei	252.83±29.52(91)	191.27±30.30(78)
*Lophuromys stanleyi*	99.2±15.82(118)	284.93±19.47(197)
*Mastomys natalansis*	66.37±14.50(66)	121.7±20.28(108)
*Lemniscomys stratus*	183±04(50)	217±16(76)
*Praomys jacksoni*	164.37±28.61(99)	83.07±11.75(150)

^*^Number in brackets represents the number of animals captured per season

Further analysis indicated that there was no significant difference in home ranges of the dominant rodent species across seasons ($${F}_{1, 10}= 0.252, p = 0.422)$$ (Table 7).

**Table 7:** Analysis of variance based on home range by season

**Source**	**SS**	D**f**	**MS**	**F**	**Prob > F**
Between groups	16.74	1	16.74	0.252	0.422
Within groups	663.61	10	66.36		
Total	680.35	11			

## Discussion

In total, 13 species were recorded from the three study sites. The regenerating forest habitat had the highest species diversity, and this could be attributed to the evident plant diversity therein. This is because habitats with high plant cover and diversity tend to have many food alternatives, thus attracting more rodent species. This observation is in agreement with the findings in many other studies done previously [13, 18]. Across those studies, it is clearly pointed out that regenerating forest habitats (Secondary forests) tend to have high diversity of plants, and they dominate most gaps created as a result of habitat destruction. This attracts more rodents due to increased food alternatives. Besides, such differences could further be explained by the ever changing human activities which modify different habitat attributes, thus impacting on rodent communities. Due to the loss of plant cover, forest and bush encroachment, changes in the small mammal community were most likely caused by the loss of food resources, disruption of habitat structures, cover and shelter and by increased predation risk due to exposure [19, 20]. The low species diversity in the depleted forest habitat, could be attributed to the frequent bush and charcoal burning within the area.

The most dominant rodent species were; *L. stanleyi*, *H.stella*, *P.jacksoni*, *M. natalensis*, *L. ansorgei*, and *L. striatus*. *L. stanleyi* was the most captured rodent specie and prefers inhabiting areas with thick vegetation (disturbed forest habitats) [21]. The dominance of *L.* *stanleyi* in the regenerating and depleted forest habitats is a sign that there might have been forests before human invasions and settlements in such areas. This finding further confirms that *Lophuromys* species are highly flexible and tend to take advantage of habitats under regeneration/changing environments or undergoing any form of transformation. *H. stella* and *P.* *jacksoni* were reported second, and third, respectively in numbers. The two species occurred mainly in the undisturbed part of the forest reserve or areas with limited levels of disturbance. This finding was not a surprise because the duo are known as forest dwellers, and tend to prefer intact forests with very limited disturbance [15]. The current result is in agreement with previous studies done in Uganda and Tanzania [22, 23]. The high number of *M. natalansis*, and *L. ansorgei*, especially in the regenerating forest habitats, confirms that the two have a relatively wider distribution compared to many African rodents, and their distributions gradually increase with increase in habitat disturbance [15]. Besides, the two species prefer thick vegetation with relatively cool environments, these where evident in the regenerating forest in form of bushed grass lands and abandoned bushed garden patches.

*L. ansorgei* and *L. striatus* were observed to have larger home ranges in the depleted forest and a lower home ranges in the intact and regenerating forest habitats, respectively. *L. stanleyi*, and *M. natalensis* had larger home ranges in the regenerating forest as compared to other habitats. In the intact forest habitat, *H. stella* and *P. jacksoni* had the larger home ranges, and relatively smaller home ranges in the regenerating forest*.* Across all habitats, it was noted that the higher the number of a particular specie in a given habitat, the larger its home range could be. This could be due to the fact that competition for scarce resources (food, nesting places, among others) always increases with increase in rodent abundance. Further analysis indicated that home ranges of the dominant rodent species significantly varied across the three habitats. This finding is consistent with the study done by [2], which noted that factors affecting resource availability and distribution directly affect home range size. Besides, home range can also vary amongst different species due to differences in the quality of habitat, distribution, and abundance of food [24].

Majority of the dominant species (*H. stella*, *L. striatus*, *L. stanleyi*, *M. natalensis*) were most dominant during the wet season. This could be attributed to the availability food alternatives, and the conducive conditions for breeding. The home ranges of the dominant rodent species did not vary depending on seasons. This could be attributed to the fact in Uganda we always have two main rainy seasons (MAM & SOND), however rains were received throughout the study period, and therefore no clear distinction among the two seasons. Thus, it was not possible to ascertain any significant variations in home ranges across seasons.

*L. ansorgei* and *P. jacksoni* had a high home range in dry and lower in wet seasons. *H. stella*, *L. stanleyi*, *M. natalensis*, and *L. striatus* had higher home ranges in the wet season and lower home ranges in the dry season. This could be premised on the fact that resources were sufficiently available throughout the time of trapping in the study area [25]. These findings concur with the findings of other scholars, including [16].

## Conclusion

Thirteen species were captured throughout the study period with high numbers of rodents captured in the regenerating forest and low numbers in the depleted forest habitat. *L. stanleyi*, *H.stella*, *P. jacksoni*, *M. natalensis*, *L. ansorgei*, and *L. striatus* were the most dominant rodent species. Overall, *L. stanleyi* was the most dominant specie and tends to dominate habitats with thick vegetation (the regenerating and depleted forest habitats). *L. ansorgei* had a larger home range size while *M. natalensis* had the lowest home range size. *L. ansorgei* had the highest home range size in the depleted forest, closely followed by *L. stanleyi*, and *L. striatus*, respectively in the same habitat. In the regenerating forest habitat, *M. natalensis* had a relatively larger home range size, followed by *L. stanleyi*, and *L. striatus*, while in the intact forest habitat, *H. stella* had the biggest home range followed by *P. jacksoni.* Further analysis indicated that home ranges of the dominant rodent species in Mabira Central Forest Reserve significantly varied across the three habitats. To the contrary, the home ranges of the dominant rodent species did not significantly vary across seasons. Thus, the rodent management strategies in disturbed forest reserves should focus most on the type of the habitat.

## Data Availability

The authors hereby confirm that the data generated and analyzed during the current study are available at a free cost from the corresponding author on request.

## Abbreviations

ACE: African Centre of Excellence
IRPM: Innovative Rodent Pest Management
BTD: Biosensor Technology Development
MCFR: Mabira Central Forest Reserve
MAM: March to May
SOND: September to December
DNA: Deoxyribonucleic acid
MCP: Minimum Convex Polygon
ANOVA: Analysis of Variance
UWA: Uganda Wildlife Athority
UNCST: Uganda National Council for Science and Technology
CMR: Capture-Mark-Recapture
SUA: Sokoine University of Agriculture
